# Explicit Preston’s Equation Describes the Geometries of Egg-Shaped Tomato Cultivars and Its Potential for Estimating the Volume and Surface Area

**DOI:** 10.3390/plants14213398

**Published:** 2025-11-06

**Authors:** Weiwei Huang, Jiaxin Tan

**Affiliations:** 1 State Key Laboratory for Development and Utilization of Forest Food Resources, Nanjing Forestry University, Nanjing 210037, China; 2 Co-Innovation Center for Sustainable Forestry in Southern China, Bamboo Research Institute, College of Forestry and Grassland, Nanjing Forestry University, Nanjing 210037, China; tanjiaxin@njfu.edu.cn; 3 Department of Geosciences and Natural Resource Management, The University of Copenhagen, Rolighedsvej 23, DK-1958 Frederiksberg, Denmark

**Keywords:** explicit Preston equation, tomato geometry, solid of revolution, scaling relationship

## Abstract

In nature, some tomato (*Solanum lycopersicum*) shapes appear to be ellipsoidal. This study aims to fit the ellipsoid tomato profile using explicit Preston’s equation (EPE), and calculate its volume (*V*_pred_) and surface area (*S*) based on the estimated EPE’s parameters. This method offers low-cost and non-destructive advantages compared to three-dimensional (3D) scanning. A total of 917 tomatoes from three cultivars were photographed, and the two-dimensional (2D) boundary coordinates of each fruit profile were digitized and then fitted using EPE. The results demonstrated that the EPE effectively fitted the tomato 2D-profile, with truss tomato ranking highest, followed by cherry, and then Qianxi. A significant relationship was found between *V*_pred_ and observed volume (*V*_obs_) at the cultivar level. The 95% confidence intervals for the slopes for cherry tomatoes include 1.0, and for Qianxi were close to 1.0, which confirmed that these two cultivars were solids of revolution. Additionally, for cherry and Qianxi tomato, *S* is proportional to the *V*_obs_ (i.e., *S*∝*V*_obs_^0.62~0.63^), *V*_pred_ is proportional to (*LW*^2^)^0.73~0.74^, and *S* is proportional to (*LW*^2^)^0.49^ (*L* is the length and *W* is the maximum width). For any isometrically scaling solid of revolution, the theoretical exponent of surface area to volume is exactly 2/3. The observed exponent of 0.62–0.63 is a biological reality, which reveals that evolution has shaped organisms not for geometric similarity, but for functional optimization. This study can be extended to a geometry study on other egg-shaped fruits and provides a potentially simple method for calculating volume and surface area based on photographed 2D fruit profiles.

## 1. Introduction

The fruit is one of the key morphological innovations associated with the explosive radiation of angiosperms [1]. In addition, fruits provide critical nutrients such as vitamins, dietary fiber, proteins, carbohydrates, and bioactive compounds like antioxidants, which are indispensable for human nutrition [1,2,3]. Through natural selection and adaptive evolution, angiosperms have developed a diverse array of fruit traits, encompassing variations in color, shape, size, and texture [4,5,6]. Therefore, the study of fruit traits can provide valuable information for understanding the mechanisms underlying the evolution of plant morphology and reproductive biology. Variation in fruit size and shape is not only the result of natural selection during domestication, but also the result of artificial selection during adaptation to different environments, cultivation methods, frugivore preferences, product storage and processing methods, among others [7]. Lomáscolo et al. [8] performed unbiased estimates of odor and color in figs and conducted comparative multivariate phylogenetic tests. They demonstrated that fruit traits evolve consistently and as predicted by behavioral, physiological (perceptual), and morphological differences in frugivorous seed dispersers. Wild cucumbers (*Cucumis sativus* var. *hardwickii*) or melons (*Cucumis melo* L.) usually bear small, rounded fruits (3–5 cm in diameter) weighing 25–35 g per fruit, while cultivated fruits can weigh up to 5 kg or 35 kg, respectively [7,9,10,11]. Fruit size is determined by coordinated cell division and expansion processes, which are directly regulated by carbohydrate allocation, water transport dynamics, and carbon metabolism within developing fruits [12]. Water deficiency typically results in late-stage fruit dropping and depresses fruit mass via decreased cell size and number [12,13]. Under intensive water deficiency, the induced carbon starvation may negatively regulate fruit cell division [14,15,16]. In previous studies, water deficiency resulted in depressed fruit growth in many species, e.g., in tomato (*Solanum lycopersicum* L.) [17,18,19], cucumber (*C. sativus*) [20], melon [21], etc. RGB images could be an idea to non-invasively determine moisture-related traits, 2D dimensions, and greenness of fruits [22,23].

Although fruit size and shape are critical factors in horticulture and developmental biology [24], many previous studies have often lacked the mathematical precision necessary to quantify these traits and provide rigorous definitions [25,26]. Fruit mass has frequently been used as a reliable indicator of ‘size’, which is evaluated by fruit length and diameter [20,21]. However, most of the fruits are not flat-shaped, but are three-dimensional (3D) approximations. Fruit volume (*V*) and surface area (*S*) are more reliable predictors of fruit size than simple fruit length and width. However, accurately measuring *V* of each fruit can be tedious, and image processing has been used to calculate *S* of fruits non-destructively, but most of the methods require the use of expensive imaging equipment, such as X-ray CT imaging, hyperspectral imaging, and 3D scanning [27,28,29,30,31]. In the realm of natural forms, the fruits of many species have good geometric symmetry and appear to be rotating solids. If a fruit conforms to being a solid of revolution, its shape can be accurately generated by revolving its two-dimensional (2D) profile by π. He et al. [25] and Wang et al. [26] demonstrated that the 2D profiles of *Cucumis melo* L. var. *agrestis* Naud. and *Canarium album* (Lour.) DC. fruits can be effectively fitted by the explicit Preston equation (EPE), which confirms that these two species’ fruits are solids of revolution. In this case, based on the estimated EPE’s parameters, the *V* and *S* of the studied fruits were calculated.

Tomato (*S. lycopersicum*) is a fruit vegetable of significant economic importance worldwide, known for its high nutritional value. In addition to its unique biochemical properties, nutrient content, short life cycle, and high seed production, the tomato has become a widely used model for research on the physiology and development of fleshy fruits [32]. The domestication and breeding of cultivated tomatoes have led to a diverse range of fruit shapes and sizes [33]. The geometry of some cultivated tomato fruits resembles that of an ellipsoid. Fruit size and shape are critical factors in horticulture and developmental biology. This study employs mathematical formulas to quantify these traits and provides the mathematical precision required for a rigorous definition. Furthermore, it utilizes mathematical formulas and 2D contour photographs captured by smartphones to explore simplified methods for calculating the volume and surface area of elliptical tomatoes. The current devices (smartphones) have demonstrated their potential for simple applications in commercial or agricultural settings. Specifically, this study aims to achieve several objectives: (1) to investigate whether EPE can effectively describe the 2D profile of tomato fruits and to determine if the tomato fruit geometry aligns with a solid of revolution; (2) to further investigate the use of equations for *V* and *S* for the solid of revolution to calculate *V* and *S*; (3) to examine if there is a statistically significant and robust scaling relationship between *S* and *V*; and (4) do *V* and *S* scale as the product of length (*L*) and maximum width (*W*) squared. To achieve this goal, we photographed 917 tomatoes from three cultivars and fitted their 2D profiles using an explicit form of Preston’s equation.

## 2. Results

**Figure 1:**
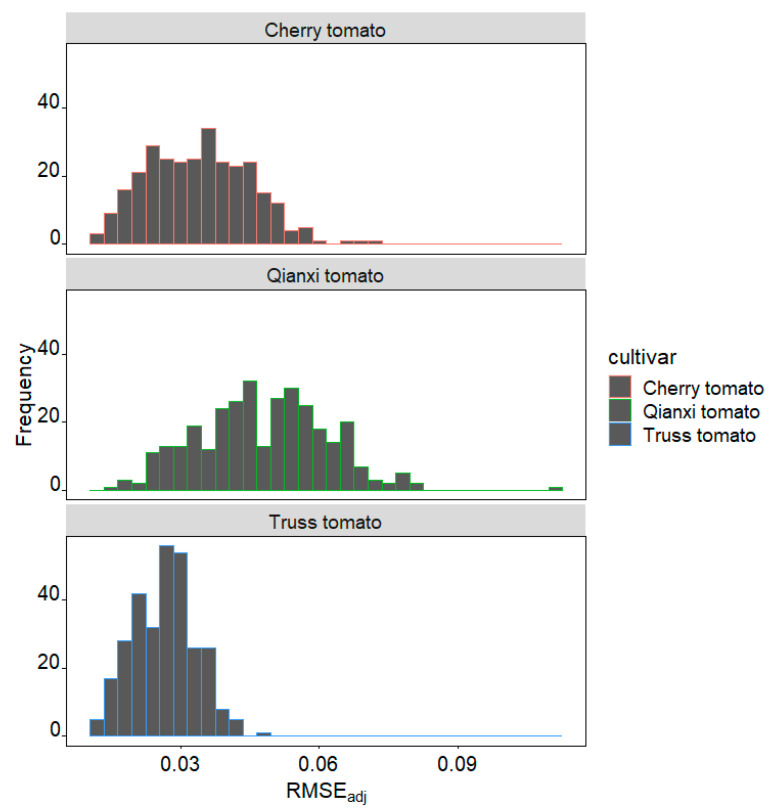
Frequency distribution of adjusted root-mean-square error (RMSE_adj_) values. The sample sizes for cherry tomatoes, Qianxi tomatoes, and truss tomatoes were 297, 320, and 300, respectively.

**Figure 2:**
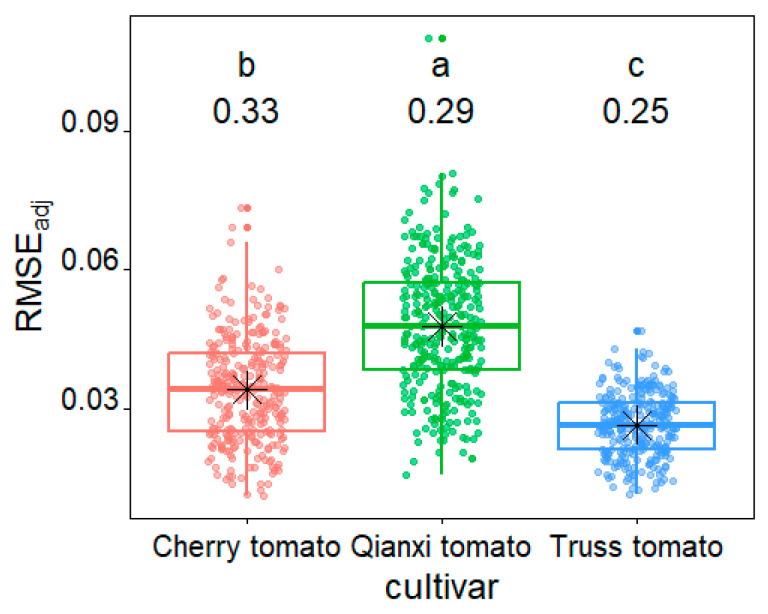
The values of RMSE_adj_ were obtained using the explicit Preston equation (EPE). Different letters indicate significant differences among the three cultivar groups (Tukey’s HSD test at the 0.05 significance level). The numbers above the box-whisker plots represent the coefficients of variation. The horizontal solid line indicates the median, the black asterisk denotes the mean, and the whiskers extend to 1.5 times the interquartile range from the top and bottom of the box. The sample sizes for cherry tomatoes, Qianxi tomatoes, and truss tomatoes were 297, 320, and 300, respectively.

**Figure 3:**
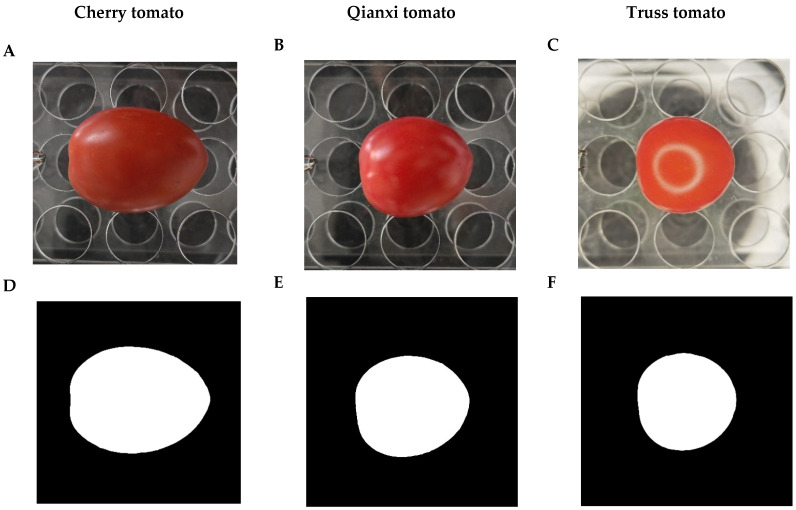

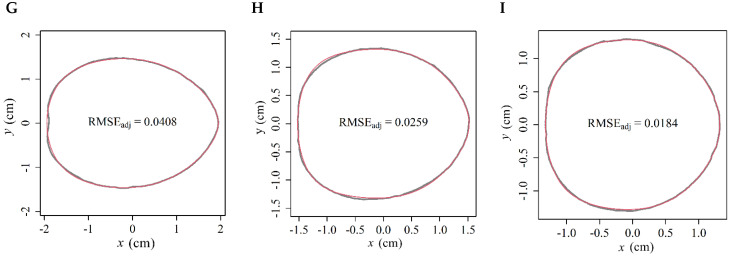
Image processing using the explicit Preston equation (EPE) to fit profiles of tomato (*Solanum lycopersicum*) from three cultivars. (**A**–**C**) Representative images of the fruits studied. (**D**–**F**) Black and white images. (**G**–**I**) The observed shapes (grey curves) and fitted shapes (red curves) of the tomato boundary for the three cultivars of tomato examples using the EPE. RMSE_adj_ represents the adjusted root-mean-square error, calculated as the RMSE between the observed and predicted *y*-values divided by half of the tomato’s maximum width. (**A**,**D**,**G**) represent the cherry tomato. (**B**,**E**,**H**) represent the Qianxi tomato. (**C**,**F**,**I**) represent the truss tomato.

The adjusted root-mean-square errors (RMSE_adj_) values of cherry tomato, Qianxi tomato, and truss tomato ranged from 0.011 to 0.073, 0.016 to 0.110, and 0.012 to 0.047, respectively, which indicates a good fit by the EPE for each tomato profile in general (Figure 1). Compared among the three cultivars studied, truss tomato showed the best fit with a median RMSE_adj_ value of 0.026, cherry tomato took the second best fit with a median RMSE_adj_ value of 0.034, and Qianxi tomato showed the worst fit with a median RMSE_adj_ value of 0.048 (Figure 2). The coefficient of variation (CV) for RMSE_adj_ across the three varieties was as follows: cherry tomato 33%, Qianxi tomato 29%, and truss tomato 25%. This indicates that the truss tomato exhibited the smallest variation in fitting error and the highest fitting stability. An example of the observed (grey lines) and predicted (red lines) tomato profiles of the three cultivars is shown in Figure 3.

**Figure 4:**
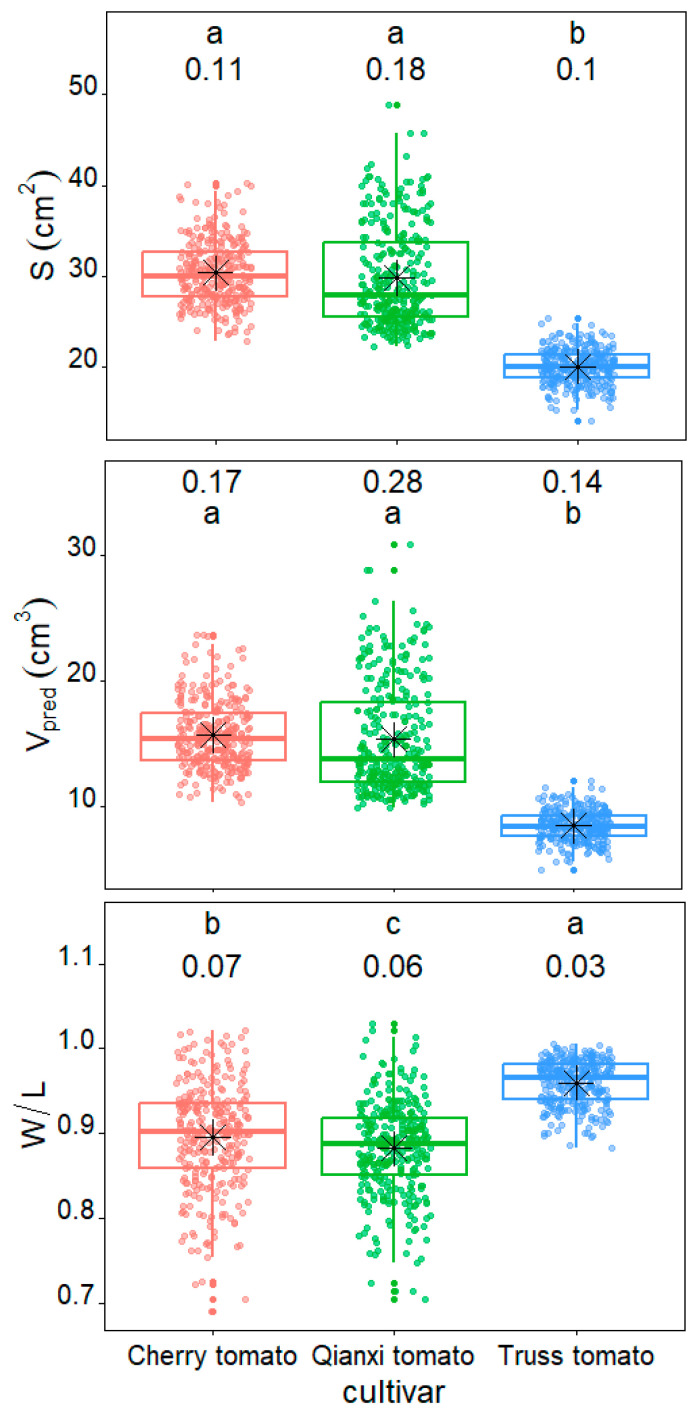
Box–whisker plots comparing the surface area (*S*), predicted volume (*V*_pred_), and the ratio of maximum width to length (*W*/*L*) for the three tomato cultivars studied. The solid horizontal line indicates the median, the black asterisk denotes the mean, and the whiskers extend to 1.5 times the interquartile range from the top and bottom of the box. Different letters indicate significant differences among the three cultivar groups, determined by Tukey’s HSD test at a 0.05 significance level. The numbers above the box–whiskers represent the coefficients of variation.

There were significant differences in both tomato size and shape among the three tomato cultivars. The *S* value ranged from 22.93 to 40.20 cm^2^, 22.28 to 48.91 cm^2^, and 14.15 to 25.45 cm^2^ for cherry tomato, Qianxi tomato, and truss tomato, respectively (Figure 4). The median *S* value of cherry tomato (*S* = 29.97 cm^2^) and Qianxi tomato (*S* = 27.92 cm^2^) was significantly higher than that of truss tomato (*p* < 0.05; *S* = 20.07 cm^2^). The predicted volume (*V*_pred_) value ranged from 10.30 to 23.67 cm^3^, 9.86 to 30.85 cm^3^, and 4.99 to 12.05 cm^3^ for cherry tomato, Qianxi tomato, and truss tomato, respectively (Figure 4). In addition, the median *V*_pred_ value of cherry (*V*_pred_ = 15.34 cm^3^) and Qianxi (*V*_pred_ = 13.77 cm^3^) tomato was also significantly higher than that of truss tomato (*p* < 0.05; *V*_pred_ = 8.43 cm^3^). Based on the value of *W*/*L*, the cherry (median *W*/*L* = 0.90) and Qianxi (median *W*/*L* = 0.89) tomatoes were more elliptical than truss tomatoes (median *W*/*L* = 0.97).

**Figure 5:**
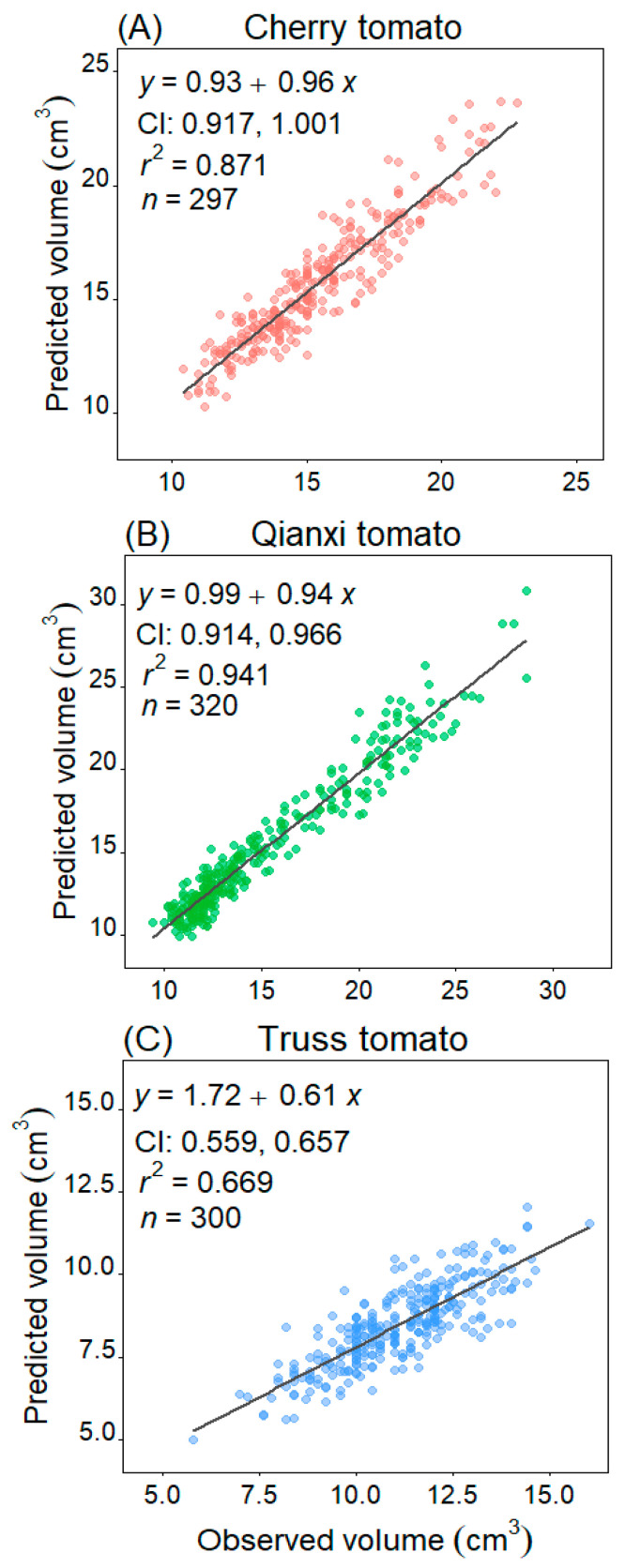
Linear regression analysis comparing the predicted volume (*V*_pred_) to the observed volume (*V*_obs_) of the three tomato cultivars. CI denotes the 95% confidence interval of the slope. The *r*^2^ value indicates the coefficient of determination, reflecting the goodness of fit, while *n* represents the sample size, which is the number of tomatoes.

A statistically significant association between *V*_pred_ and observed volume (*V*_obs_) for the three tomato cultivars was found (*p* < 0.05). Specifically, Qianxi tomato showed the best relationship between *V*_pred_ and *V*_obs_ with *r*^2^ of 0.941, cherry tomato took the second place with *r*^2^ of 0.871, and truss tomato had the lowest *r*^2^ of 0.669. The 95% CIs of the slope of *V*_pred_ vs. *V*_obs_ include the value 1.0 for the cherry tomato studied. Although the 95% CIs of the slope for Qianxi tomato ranged from 0.914 to 0.966 and did not include 1.0, the *r*^2^ was 0.941, and the slope is close to 1.0 (Figure 5). This finding supports the hypothesis that the cherry and Qianxi tomato cultivars studied are considered as solids of revolution.

**Figure 6:**
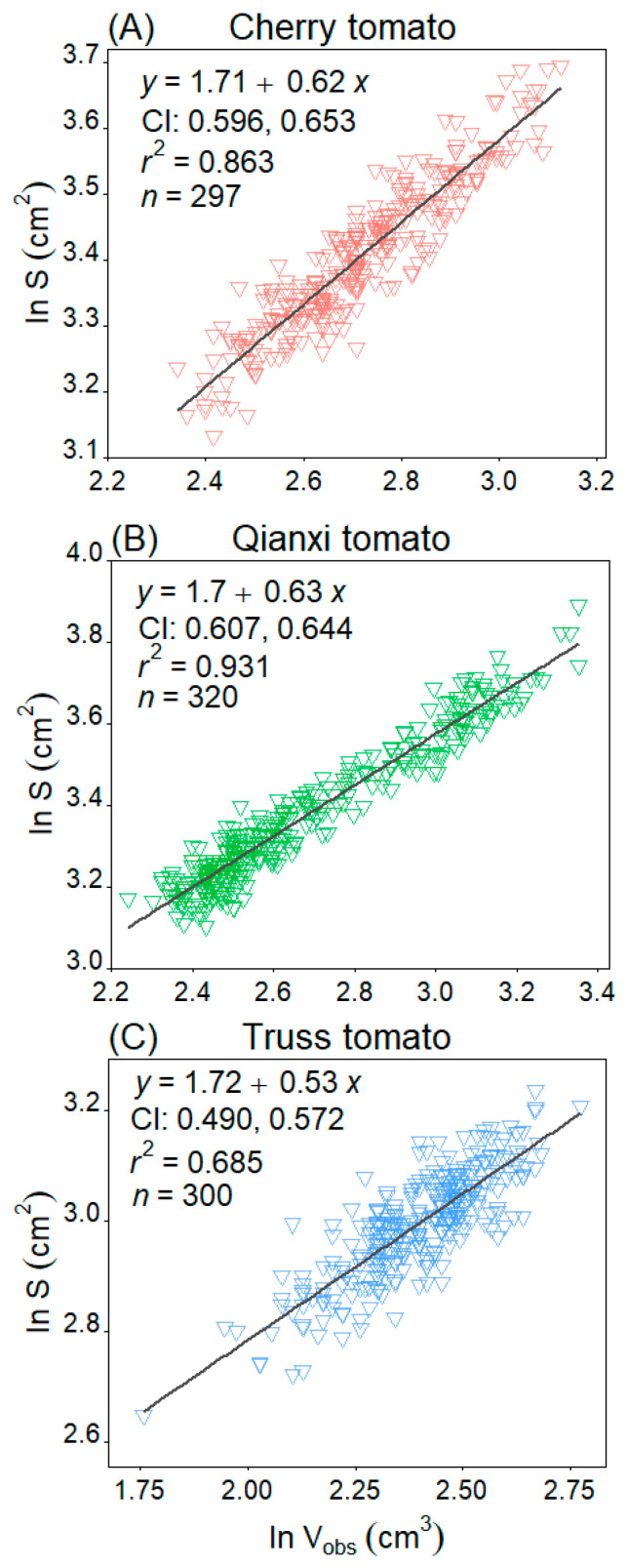
Linear regression analysis of the surface area (*S*) and observed volume (*V*_obs_) of the three tomato cultivars on a log–log scale. CI represents the 95% confidence interval of the slope. The *r*^2^ value is the coefficient of determination, and *n* represents the sample size, which refers to the number of tomatoes.

**Figure 7:**
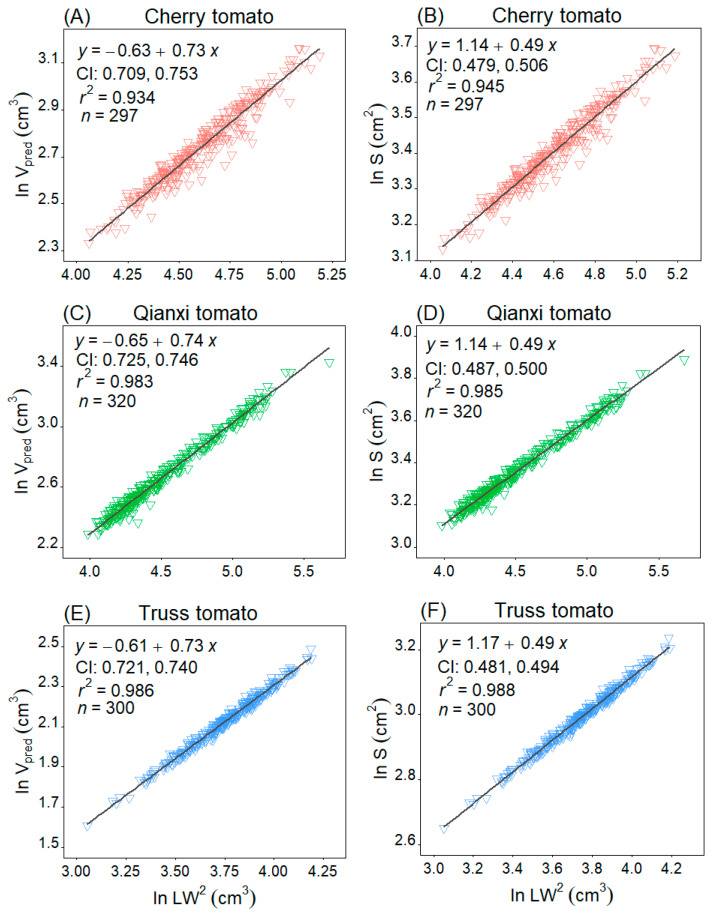
Fitted scaling relationships between predicted volume (*V*_pred_) and the product of length (*L*) and maximum width (*W*) squared (i.e., *LW*^2^), and between surface area (*S*) and *LW*^2^. CI represents the 95% confidence interval of the slope. The *r*^2^ value is the coefficient of determination, and *n* represents the sample size, which refers to the number of tomatoes.

A significant scaling relationship was found between *S* and *V*_obs_ for the three tomato cultivars (*p* < 0.05). Specifically, Qianxi tomato showed the best relationship between *S* and *V*_obs_ with *r*^2^ of 0.931, cherry tomato took the second place with *r*^2^ of 0.863, and truss tomato had the lowest *r*^2^ of 0.685 (Figure 6). In addition, the *V*_pred_ versus *LW*^2^ and *S* versus *LW*^2^ are statistically robust (Figure 7), and *V*_pred_ is proportional to (*LW*^2^)^0.73~0.74^, and *S* is proportional to (*LW*^2^)^0.49^.

## 3. Discussion

### 3.1. Validity of EPE in Modeling Three Tomato Cultivars

Preston [34] proposed an elliptic parametric equation to model avian egg shapes, a framework now widely employed to analyze the 2D profiles of eggs across diverse bird species [35]. Preston’s equation (PE) can be estimated using multiple linear regression protocols. However, PE cannot provide an explicit relationship between the vertical (*y*) and horizontal (*x*) coordinates of an egg’s empirical profile. Furthermore, the PE is based on the assumption of perfect bilateral symmetry, which may not hold for many organic structures, such as tomatoes. Based on the modified form of Preston’s equation introduced by Todd and Smart [36], Shi et al. [35] developed a nonlinear optimization algorithm to estimate the parameters for the explicit Preston equation [EPE, Equation (1)], incorporating considerations for fruit profile asymmetry. This explicit expression relating *y* and *x* can directly calculate the volume (*V*) and surface area (*S*) of a *C. melo* var. *agrestis* and *C. album* fruit [25,26]. In the current study, based on the empirical measurement of 917 tomatoes, the data validate the predictions of EPE, with RMSE_adj_ values of cherry tomato, Qianxi tomato, and truss tomato ranging from 0.011 to 0.073, 0.016 to 0.110, and 0.012 to 0.047, respectively. Of the three cultivars studied, truss tomato had the best fit with RMSE_adj_ less than 0.05 in 100% of studied fruits, cherry tomato took the second best fit with RMSE_adj_ less than 0.05 in 92%, and Qianxi tomato had the worst fit with RMSE_adj_ less than 0.05 in 54% (Figure 1 and Figure 2). Deformed by a variety of abiotic and biotic factors, the surface of fruit is usually not sufficiently smooth, and its 3D geometry is also not as regular as that of an egg [26,35], resulting in profiles that are not perfectly bilaterally symmetrical. The nonlinear optimization method was employed effectively to address the issue of asymmetric fruit contours [35,37]. For example, He et al. [25] and Wang et al. [26] used the EPE to fit *C. melo* var. *agrestis* and *C. album* fruits and found that all the RMSE_adj_ values for the 751 *C. melo* var. *agrestis* fruits and 574 *C. album* fruits are below 0.05. The surface of truss tomato, *C. melo* var. *agrestis* and *C. album* fruits is smoother compared to cherry tomato and Qianxi tomato (Figure 3), which may have contributed to their 2D profile better fitting the EPE. Future research may integrate RGB imaging or hyperspectral imaging techniques to extract color indices from fruit surfaces. This approach will enable further analysis of the relationships between fruit morphological variation, physiological status, and environmental adaptability, thereby providing a more comprehensive assessment of fruit quality and stress resistance.

### 3.2. Are the Three Tomato Cultivars Studied Agrestis a Rotating Solid?

A solid revolution is a 3D geometric shape formed by rotating a planar curve around a straight axis. If the 2D profile of a 3D geometry is bilaterally symmetric with respect to its centerline, i.e., the longest line segment connecting the two ends of the profile coincides with the axis of rotation, then rotating the geometry around the centerline by any angle does not affect its 2D projected area, and the geometry is a rotated solid. Assuming that the tomato fruit is a rotating solid, its volume and surface can be estimated using rotational geometry equations and compared to the observed volume (*V*_obs_) measured via water displacement. The volume of cherry tomato [38], watermelon (*Citrullus lanatus* cv. ‘Sueme’) [39], tangerine (*Citrus reticulata*) [40], and Orange (*Citrus Aurantium*) [41] estimated by image processing was not significantly different from volumes obtained using water displacement. In this study, cherry tomato and Qianxi tomato exhibited a strong correlation between *V*_pred_ and *V*_obs_ (slopes 95% CIs include 1.0 for cherry; slopes 95% CIs close to 1.0 for Qianxi), with median relative error of 4.370% and 4.955%, respectively. These errors fall within the acceptable 5% range reported in previous fruit volume studies (such as lemons, limes, oranges, tangerines, and tomatoes) [38,42]. Truss tomato had a relatively poor correlation (slopes 95% CIs 0.559–0.657, *r*^2^ = 0.669). Two hypotheses may explain this difference. Firstly, smaller truss tomatoes relative to the cylinder diameter potentially increased water displacement measurement error [35]. Secondly, although truss tomato exhibits a good 2D profile fit by EPE, its nearly round 3D shape (*W*/*L* approaching one) does not correspond to a rotating solid so well, and the volume and surface area equations for rotating solids do not allow for a good estimation. Furthermore, methodological consistency in image acquisition is crucial. Implementing defined illumination conditions and a fixed sample position during photographing would improve the reproducibility of the 2D profile extraction and might help reduce such discrepancies in future studies.

### 3.3. Can These Results Be Extended to Other Fruit Species and Used in Future Tomato Fruit Size Measurements?

Visa et al. [43] used elliptic Fourier shape modeling to model the tomato fruits into nine shape categories: round, rectangular, ellipsoid, flat, obovoid, oxheart, long rectangular, heart, and long. The current study confirms that the EPE was verified to fit the 2D profile of three near-egg-shaped tomato cultivars. The EPE showed potential for describing the 2D profiles of other egg-shaped fruits. Future work may explore applying additional mathematical models to fit the 2D profiles of other fruits with regular profiles (e.g., citrus, persimmon, pepper, eggplant, etc.). In addition, as shown here and previously, the smoother the profile is, the better the EPE fitted [25,26]. Looking at it from another angle, fruits are subjected to the influence of their surroundings during growth, and the greater the environmental stress, the greater the irregularity in fruit shape. Measuring the deviation of fruit geometry from the standard ellipsoid can provide insights into the morphology of the plant and how it is affected by environmental conditions, and to a certain extent, its evolution. Applying this approach across different developmental stages could provide valuable insights into the dynamics of fruit growth and shape ontogeny.

Real-time tomato surface area and volume measurement is laborious [38]. This study showed Qianxi and cherry tomato can be accurately modelled as a solid of revolution, enabling the estimation of volume and surface area using rotational geometry equations. A strong *V*_obs_-*S* correlation was confirmed [26], with Qianxi (*r*^2^ = 0.931) and cherry (*r*^2^ = 0.863) showing better fits compared to truss tomato (*r*^2^ = 0.685). This may be because the 3D shape of the truss tomato does not coincide with the rotating solid, and the volume and surface area estimated from equations for the rotating solids are less than optimal. Further, the results showed that the *S* of an egg-shaped tomato scales approximately at 0.62–0.63 times its volume (i.e., *S*∝*V*^0.62~0.63^, Figure 6A,B). The *V*_pred_ versus *LW*^2^, and *S* versus *LW*^2^ scaling relationships of the egg-shaped cultivars mentioned were statistically robust, and *V* is proportional to (*LW*^2^)^0.73~0.74^ and *S* is proportional to (*LW*^2^)^0.49^ (Figure 7). Thus, this enables the development of an efficient method for calculating egg-shaped fruit volume and surface area.

The practical applications of this method may extend to multiple fields. Plant researchers can use it for precise morphological studies. Breeders can use the parameters for non-destructive selection. In agriculture, it may enable low-cost volume estimation for grading. Furthermore, it serves as an excellent model for teaching geometrical concepts. The methodology presented here holds commercial potential due to its inherently low-cost and scalable nature. By relying on standard smartphone cameras and freely available R packages (e.g., biogeom, version 1.3.5), the barrier to entry is minimal. The image analysis and EPE fitting (or other mathematical fitting) pipeline may be embedded into a mobile application for use in the field by breeders and farmers, or integrated into the camera systems of automated grading lines in packhouses. Such a system may enable non-destructive, high-throughput grading of fruit based on precise geometric attributes like volume and surface area, which are key indicators of yield and market value. This may offer a practical and affordable solution for breeders seeking to phenotype large populations, for packhouses aiming to sort fruit efficiently, and for retailers requiring consistent quality control, moving beyond simplistic size grading to more sophisticated shape and volume-based classification.

## 4. Materials and Methods

### 4.1. Sampling

Three cultivars of tomatoes were selected for this study, including cherry tomato, Qianxi tomato, and truss tomato. A total of 917 mature and undamaged tomatoes were sampled (297 for cherry tomato, 320 for Qianxi tomato, and 300 for truss tomato). All fruits were harvested at the mature ripening stage, characterized by a fully developed red color and firm texture, to ensure physiological and geometric comparability. The sampled cherry tomatoes were growing in Baise, Guangxi, China, Qianxi tomatoes were growing in Liaocheng, Shandong, China, and truss tomato plants were growing in Pingliang, Gansu, China, and collected in November 2024, November 2024, and December 2024, respectively. After harvesting, tomatoes were transported to the laboratory under refrigerated conditions. Upon arrival, measurements were completed as soon as possible that same day while maintaining refrigerated storage to prevent dehydration. A representative image of the three studied tomato cultivars is shown in Figure 3.

### 4.2. Photographing and Data Acquisition

Each studied tomato was photographed horizontally using a smartphone (Honor Magic 4, Shenzhen, China) mounted on an adjustable tabletop phone stand, positioned horizontally, and fixed at a consistent height. The tomato was positioned horizontally with the pedicel end on the left and the blossom end on the right, and stabilized with a test tube holder (Figure 3A–C). We adjust each tomato’s central axis to be approximately parallel to the surface of the desk by observing whether the straight line through the fruit base and apex is approximately parallel to the desk’s surface. The length of each tomato was measured using a vernier caliper (accuracy: 0.02 mm) to calibrate image dimensions. Photographs were converted to black-and-white images using Adobe Photoshop CS2 (version 9.0; Adobe, San Jose, CA, USA) and saved as bitmap images at 600 dpi, in order to obtain the planar coordinates of the tomato 2D profile (Figure 3D–F). The in-plane coordinates of each tomato 2D profile were extracted from the corresponding BMP black-and-white images using the program developed by Shi et al. [44] and Su et al. [45] on Matlab (version ≥ 2009a; MathWorks, Natick, MA, USA). Based on the horizontal length of tomatoes measured using a vernier caliper, the black-and-white images underwent size modification. Specifically, the images were scaled proportionally according to the actual measured lengths to adjust their dimension. The “adjdata” function in the “biogeom” package (version 1.3.5) in R software was employed to digitize the profile of each studied tomato [46]. The number of approximately equidistant data points forming each tomato profile was fixed at 1000.

### 4.3. Data Fitting and Modelling

The mathematical expression for the explicit Preston equation (EPE) is as follows:

(1) $$y\;=\;\pm a\sqrt{1-\;{\left(\frac{x}{b}\right)}^{2}}\left(1+c_{1}\left(\frac{x}{b}\right)+{c_{2}\left(\frac{x}{b}\right)}^{2}+{c_{3}\left(\frac{x}{b}\right)}^{3}\right)$$

where *x* and *y* are the horizontal and vertical coordinates in the Cartesian plane, *a* and *b* are approximately half of the tomato’s maximum width and half of the tomato’s length, respectively (in cm). The plus and minus signs on the right side of Equation (1) represent the upper and lower portions of the tomato profile, where the lines are aligned along the *x*-axis. The “fitEPE” function in the “biogeom” package (version 1.3.5) was performed to fit the explicit Preston equation and estimate the numerical values of *a*, *b*, *c*_1_, *c*_2_, and *c*_3_ [46]. The parameters of the explicit Preston equation were estimated using the Nelder-Mead optimization method to minimize the residual sum of squares between the observed and predicted *y*-values of tomato profiles [47].

Assuming that the tomato fruit is a rotating solid, its volume can be estimated using the volume and surface area equations for rotating solids to estimate the predicted volume (*V*_pred_) and surface area (*S*) of its fruit. The volume and surface area equations are

(2) $$\begin{array}{c} V=\pi \int_{-b}^{b}y^{2}dx\\ \;\;\;=\frac{4}{315}\pi a^{2}b\left(105+21c_{1}^{2}+42c_{2}+9c_{2}^{2}+18c_{1}c_{3}+5c_{3}^{2}\right) \end{array}$$

and

(3) $$S\;=2\pi \int_{-b}^{b}y\sqrt{1+{\left(\frac{dy}{dx}\right)}^{2}}dx$$

where *a* is approximately half of the tomato’s maximum width, *b* is half of the tomato length [(as in Equation (1)], and $\frac{dy}{dx}$ is the derivative of *y* with respect to *x*.

In addition, the observed volume (*V*_obs_) value of each tomato was measured by submerging it in water in a 250 mL graduated cylinder with a diameter of 4 cm and recording the displacement of the liquid.

To evaluate the accuracy of the model fitting, the adjusted root-mean-square errors (RMSE_adj_) were calculated [26,48,49,50]:

(4) $${RMSE}_{adj}=\frac{\sqrt{\frac{1}{n}\sum_{i=1}^{n}{\left(y_{i}-{\hat{y}}_{i}\right)}^{2}}}{W/2}$$

where *n* represents the number of data points on a tomato profile, $y_{i}$ and ${\hat{y}}_{i}$ are the observed and predicted *y* values, respectively. *W* represents the maximum width of a tomato. As a rule of thumb, an RMSE_adj_ value of 0.05 typically indicates a good fit [26], i.e., the mean absolute deviation between the observed and predicted distances from the data point on the tomato profile to the midline of the profile is less than 5% of half of the maximum width of the tomato.

### 4.4. Data Analysis

Tukey’s Honestly Significant Difference (HSD) test was conducted at the 0.05 significance level to assess significant differences in RMSE_adj_, predicted surface area (*S*), predicted volume (*V*_pred_), and the ratio of maximum width to length (*W*/*L*) among the three cultivars. The distribution of the residuals was examined based on the values predicted by the one-way ANOVA (Analysis of Variance) model, and the results showed that the residuals were uniformly distributed. To evaluate the normality of RMSE_adj_, *S*, *V*_pred_, and *W*/*L*, normal Q-Q plots were created, and the results indicated that these four parameters closely followed a normal distribution. Additionally, to determine whether the explanatory variables influenced the dispersion of the residuals, we examined whether the dispersion of the residuals increases at a given predicted value. The findings confirmed that the basic condition of homoscedasticity for the four variables was met.

A linear regression analysis was conducted to examine the relationship between the *V*_obs_ of tomatoes and their *V*_pred_. This analysis involved comparing the predicted surface area (*S*) and *V*_obs_ of tomatoes on a log–log scale using a linear regression approach. The “confint” function in the “stats” package (version 3.6.2) was used to calculate the 95% confidence interval (CI) for each coefficient in the linear model. If the 95% CI of the slope includes the value 1.0, this indicates a statistically significant association between *V*_obs_ and *V*_pred_. Therefore, the tomatoes can be treated as solids of revolution.

## 5. Conclusions

Using morphometric data derived from 917 tomatoes of three cultivars, we show that the EPE effectively describes the 2D profile of the egg-shaped tomato, with the fit ranking as follows: truss tomato > cherry tomato > Qianxi tomato. The tomato volume (*V*_pred_) estimated using the rotating solids equation based on the estimated EPE’s parameters was compared to the observed volume (*V*_obs_) using water displacement to determine whether the tomatoes are solids of revolution. The data show that Qianxi tomatoes and cherry tomatoes can be modeled as a solid of revolution. In addition, a robust *S* versus *V*_obs_ log–log linear relationship exists, and *V*_pred_ and *S* are also shown to be proportional to the product of length and maximum width squared for these two cultivars (i.e., *V*_pred_∝(*LW*^2^)^0.73~0.74^, *S*∝(*LW*^2^)^0.49^). These methods and results provide further insights into a geometry study on other egg-shaped fruits, and the volume and surface area calculation method presented here is potentially transferable to other fruits with similar geometries. Furthermore, by employing standardized imaging protocols—including controlled illumination, fixed orientation, and standard RGB cameras—image analysis and mathematical fitting processes may be embedded into a mobile application. This may enable non-destructive, high-throughput fruit grading based on precise geometric attributes such as volume and surface area.

## Data Availability

The original contributions presented in this study are included in the article/Supplementary Materials. Further inquiries can be directed to the corresponding author.

## Supplementary Materials

The following supporting information can be downloaded at: https://www.mdpi.com/article/10.3390/plants14213398/s1, Table S1: Summary of the tomato samples.

## Abbreviations

The following abbreviations are used in this manuscript:

3D	Three-dimensional
*V*	Volume
*S*	Surface area
*V*_pred_	Predicted volume
*V*_obs_	Observed volume
*W*	Maximum width
*L*	Length
RMSE_adj_	Adjusted root-mean-square errors
EPE	Explicit Preston equation
