# Supplementary material for: Explicit Preston’s Equation Describes the Geometries of Egg-Shaped Tomato Cultivars and Its Potential for Estimating the Volume and Surface Area
Source: Plants (Basel). 2025 Nov 6;14(21):3398. doi: 10.3390/plants14213398 (PMC12609381; doi:10.3390/plants14213398)
Supplement: Supplementary file 1 [file plants-14-03398-s001.zip › Supplementary table S1.pdf]

Table S1. Summary of the tomato samples.

Cultivar	Length (cm)	Width (cm)	2D Area (cm^2)	V_{obs} (cm^3)
Cherry tomato	3.35 ± 0.24	2.99 ± 0.19	7.84 ± 0.89	15.39 ± 2.63
Qianxi tomato	3.34 ± 0.33	2.94 ± 0.27	7.72 ± 1.42	15.26 ± 4.40
Truss tomato	2.61 ± 0.12	2.51 ± 0.14	5.08 ± 0.47	11.09 ± 1.62
